# Neuromuscular contributions to inter-limb quadriceps strength asymmetries across contraction types in healthy adults

**DOI:** 10.1007/s00421-026-06251-4

**Published:** 2026-05-16

**Authors:** Hadrien Alaux, Simon Barrué-Belou, Pierre-Marie Matta, Julien Duclay

**Affiliations:** 1 Physiotherapy Department and Motion Analysis Lab, Swiss Olympic Medical Center, La Tour Hospital, Meyrin, 1217 Switzerland; 2 Université de Toulouse, CNRS, CerCo, Toulouse, France; 3 Université de Toulouse, INSERM, ToNIC, Toulouse, France

**Keywords:** Voluntary activation, Doublet-evoked torque, Electromyography, Knee extensor muscles, Anisometric contractions, Imbalance.

## Abstract

**Purpose:**

This study investigated the contribution of neuromuscular mechanisms to quadriceps strength asymmetry across isometric, concentric, and eccentric contractions by examining the covariation between strength asymmetry and asymmetries in voluntary activation and doublet-evoked torque.

**Methods:**

Fifteen healthy participants performed maximal isometric and anisometric contractions (30°/s) of both lower limbs on an isokinetic dynamometer. Side-to-side differences in torque, electromyographic (EMG) activity, voluntary activation, and doublet-evoked torque were assessed by comparing the stronger and weaker limbs. Associations between asymmetries in torque, voluntary activation, and doublet-evoked torque were subsequently examined.

**Results:**

A significant difference between the stronger and weaker limbs was observed for torque across all contraction types (162.1 and 145.6 N.m, respectively), whereas no between-limb differences were found for neuromuscular parameters. Covariation analyses indicated that asymmetries in activation level (estimate = 0.57, *p* < 0.001) and doublet-evoked torque (estimate = 0.29, *p* = 0.006) were significant predictors of torque asymmetry, together explaining 29% of its variance (Marginal R²). In contrast, these relationships were not influenced by contraction type (*p* = 0.670).

**Conclusion:**

In healthy individuals, strength asymmetries covary positively with neuromuscular asymmetries. Given the absence of contraction-type effects, findings derived from isometric conditions likely generalize to anisometric contractions.

## Introduction

Inter-limb strength asymmetry is defined by a difference in strength between one limb and the other. Such asymmetries may impair performance (Coratella et al. 2018) and increase the risk of injury (Bourne et al. 2015). Nevertheless, findings remain conflicting, as some studies have reported no significant association between strength asymmetry and performance (DosʼSantos et al. 2018). Furthermore, these asymmetries may be functional and related to sport-specific adaptations (Kalata et al. 2020) and their impact on injury risk remains debated (Opar et al. 2015). Similarly, findings regarding strength asymmetries in healthy individuals are highly heterogeneous. While certain studies demonstrate strength asymmetries in eccentric and concentric conditions measured via isokinetic dynamometry (Jones and Bampouras 2010; Thomas et al. 2017) others do not observe significant differences under the same conditions (Daneshjoo et al. 2013; González-Ravé et al. 2014). This variability may be partly attributed to factors such as biological variables (age, sex) (Carabello et al. 2010) or measurement conditions (angle of measure, contraction type) (Kocak et al. 2023), which can influence the detection of these asymmetries. Methodological choices, such as the selection of the reference limb, the calculation method, and the determination of the significance of asymmetry, could also influence the results. Firstly, in healthy subjects, various reference limbs can be employed to assess asymmetries, such as dominant/non-dominant (Cheung et al. 2012), right/left (Benjanuvatra et al. 2013), or stronger/weaker (Bishop et al. 2019). Additionally, twelve different calculation indices for strength asymmetries have been reported in the literature (Parkinson et al. 2021). Subsequently, once a strength asymmetry is calculated, threshold values of 10–15% are commonly used to determine its clinical relevance (Rohman et al. 2015; Kyritsis et al. 2016). However, the use of predefined thresholds can be problematic, as they do not account for the intra-individual variability associated with each variable tested (Bishop 2021).Therefore, it has been proposed that an asymmetry should be considered meaningful at the individual level when its magnitude exceeds the variability of the measurement, commonly expressed using the coefficient of variation (CV) (Exell et al. 2012) or when it exceeds the minimal detectable change (MDC) derived from the standard error of measurement. Taken together, this heterogeneity in the populations studied and the calculation methods employed makes it difficult to consolidate data to determine whether healthy individuals exhibit lower-limb strength asymmetries.

Despite these variabilities in measurement methodologies, investigating the underlying causes of significant strength asymmetry can provide valuable insights into the neuromuscular mechanisms involved and guide targeted rehabilitation strategies, especially when correction is desired (Bishop et al. 2018). The neuromuscular mechanisms potentially involved include structural differences in the peripheral muscle system and variations in neural activation (Lepley et al. 2020). The involvement of neural activation can be accurately evaluated through voluntary activation measurements obtained via the interpolated twitch technique (Allen et al. 1995) or surface electromyography (Vigotsky et al. 2017). This evaluation has primarily been conducted in patient populations, particularly in individuals following anterior cruciate ligament (ACL) rupture, where deficits in voluntary activation (Lepley et al. 2015; Trees et al. 2016) and electromyographic (EMG) activity (Drechsler et al. 2006) have been demonstrated. In contrast, while fewer studies exist, similar activation levels have been observed between the dominant and non-dominant limbs in healthy individuals (De Ruiter et al. 2010; Pietrosimone et al. 2012). Consistent with these findings, there are no significant differences in EMG activity between the dominant and non-dominant limbs in healthy subjects under isometric conditions, for the quadriceps muscle (Yılmaz and Kabadayı 2022) and the tibialis anterior and plantar flexor muscles (Petrovic et al. 2021). A complementary approach to understanding the neuromuscular mechanisms underlying strength asymmetries would involve examining the covariations between strength asymmetries and neuromuscular parameters. Using this approach, the contribution of activation level to torque asymmetries has been demonstrated during isometric contractions in a mixed population of healthy subjects and patients with ACL tears (Maffiuletti et al. 2016). In addition to neural activation, peripheral factors such as structural differences within the musculotendinous system and excitation-contraction coupling may also contribute to the presence of strength asymmetries (Norte et al. 2018). An involvement of excitation-contraction coupling in strength asymmetries has been demonstrated by studying the mechanical response induced by peripheral electrical stimulation of the motor nerve after a maximal contraction (doublet-evoked torque) (Maffiuletti et al. 2016). Together, these findings suggest that central neural activation and peripheral muscle factors may both contribute to the presence of strength asymmetries.

In the literature most studies examining the relationship between strength asymmetry and the involved neuromuscular mechanisms are obtained during voluntary isometric contractions. Although more accessible to implement methodologically, this approach has limitations, as strength asymmetries are, for example, commonly measured under concentric and eccentric conditions during the final phase of rehabilitation to assess patients’ strength recovery (Xergia et al. 2013). Therefore, measuring the neuromuscular parameters in concentric and eccentric conditions under the same conditions as strength measurement offers a more ecologically valid approach to understanding their implications in strength asymmetries but require further investigations. The present study therefore aimed to quantify, in healthy individuals, the contribution of neuromuscular mechanisms - including excitation-contraction coupling and neural activation - to inter-limb strength asymmetries during isometric, concentric, and eccentric contractions. A secondary aim was to determine whether the involvement of these mechanisms differs across contraction types, given the known contraction type-dependence of neuromuscular parameters (Duclay et al. 2008; Enoka and Duchateau 2016). We hypothesized that, because neuromuscular properties differ between anisometric and isometric contractions, the relative contribution of peripheral and neural mechanisms to strength asymmetries would vary according to contraction type.

## Methods

### Subjects

Fifteen healthy volunteers (15 men, age: 22 ± 3 years, mass: 74 ± 10 kg, means ± SD) who reported regular physical activity and had no history of injury participated in this study. Given that previous work reported no clear sex effect on quadriceps strength asymmetries (Lisee et al. 2019), we restricted our sample to men to ensure homogeneity. Nevertheless, future studies including women are needed to confirm our findings, particularly for the neuromuscular parameters that were not assessed by Lisee et al. (2019). All procedures complied with the Declaration of Helsinki and written informed consent was obtained from all participants after they received detailed information about the experimental protocol. The study protocol was approved by the French Ethical “Comité de Protection des Personnes Ile de France VI” (number 2022-A00032-41). The required sample size for the assessment of voluntary activation was estimated from previously reported effect sizes for contraction type (effect size = 0.66) (Krishnan et al. 2015) and limb comparisons (average effect size = 0.70) Trees et al. 2016). Assuming a statistical power (1 − β) of 0.80 and a significance level (α) of 0.05, a total sample size between 8 and 17 participants was deemed adequate for a repeated-measures ANOVA design.

### Experimental protocol

Subjects completed an experimental session lasting three hours. During the session, subjects were seated on an isokinetic ergometer (Biodex, Shirley, NY) with both knees and hips flexed at 90° (0° = full extension). The testing leg was firmly attached to the ergometer accessory at the ankle. Participants were fastened to the ergometer by two shoulder harnesses and one abdominal harness to minimize body movements and to limit the contribution of other muscle groups aside from the knee extensors. A standardized 10 min warm-up, consisting of approximately 20 submaximal isometric contractions on each leg, with progressively increasing load up to 80% of their estimated maximal voluntary torque was performed before the experimental phase. The subjects performed 24 maximal voluntary knee extensions with one leg, followed by 24 with the other leg, for a total of 48 extensions. For each leg, they completed eight repetitions of each contraction type: isometric, lengthening, and shortening. The order of leg limbs (right and left) and contraction types were randomized to prevent bias due to fatigue.

Each maximal contraction was separated by 1-min rest periods. The isometric contractions were performed at an angle of 30° of knee flexion (0° = full extension). Shortening and lengthening contraction measurements were taken at 30°s^− 1^ constant velocity over an 80° knee range of motion from 0° (full extension) to 80° of knee flexion. A 1-second isometric pre-activation was performed prior to the maximal concentric contractions at an angle of 80° of flexion, and at 0° for eccentric contractions. To encourage the subjects to achieve their maximal torque, visual feedback (instantaneous torque-time traces) was displayed on a monitor screen 150 cm in front of them, accompanied by verbal encouragement. For each contraction type, eight trials were performed under three separate conditions: (1) in three trials no stimulations were applied. (2) In two trials, double percutaneous stimulations separated by 10 ms were delivered to the femoral nerve during maximal contraction to evoke a superimposed doublet response. In these trials, the same double stimulations were also applied at rest, 3 s after contraction, to evoke a potentiated doublet. (3) In three trials, single percutaneous stimulations of the femoral nerve were applied during contraction to evoke the compound muscle action potential (M-wave). Details on the compound muscle action potential (M-wave) can be found in the EMG section. For isometric contractions, the neurostimulation was manually delivered when the torque plateaued, approximately 2 s after the start of the contraction. Subjects were asked to hold the maximal contraction for another 2 s after the superimposed stimulation. For dynamic contractions, neurostimulation was automatically delivered at a knee flexion angle of 30°, corresponding to the position used during isometric contractions. At rest, the stimulations were manually triggered, 3s after the isometric contraction and automatically at a knee flexion angle of 30°, during passive shortening (30°/s) and lengthening (30°/s) actions.

### Measurements

#### Torque and EMG

Torque was recorded at 2000 Hz using a Biopac MP150 system and Acknowledge software. Simultaneously we recorded the EMG activity (common mode rejection ratio CMRR > 110 dB, gain: 500, bandwidth: 10–500 Hz) at a sampling frequency of 2000 Hz. In accordance with the SENIAM recommendations (Hermens et al. 2000) we first prepared the skin to obtain a low impedance (< 5 kΩ). Then three pairs of electrodes (AG-Cl, diameter: 11 mm, interelectrode distance: 2 cm) were placed on the muscle belly of Vastus Medialis (VM), Vastus Lateralis (VL), and Rectus Femoris (RF). If needed, electrodes placements were adjusted to obtain the greatest M-wave amplitudes. The reference electrode was placed on the patella of the contralateral knee.

#### Motor nerve stimulation

Percutaneous stimulations were applied on the femoral nerve by a constant current stimulator (pulse duration: 500 µs; Digitimer stimulator, model DS7A, Hertfordshire, UK). The cathode (AG-Cl, 11 mm diameter) was positioned on the femoral triangle, and the anode (5 cm × 10 cm, Medicompex SA, Switzerland) was placed midway between the right great trochanter and the iliac crest. The optimal stimulation intensity was determined individually, as the minimum current that elicited maximum quadriceps twitch amplitude at rest. To ensure a supra-maximal stimulus, the intensity was then increased by 20% (Neyroud et al. 2014; Vagg et al. 1998).

### Data analysis

#### Torque, voluntary activation, and resting doublet-evoked torque

**Fig. 1:**
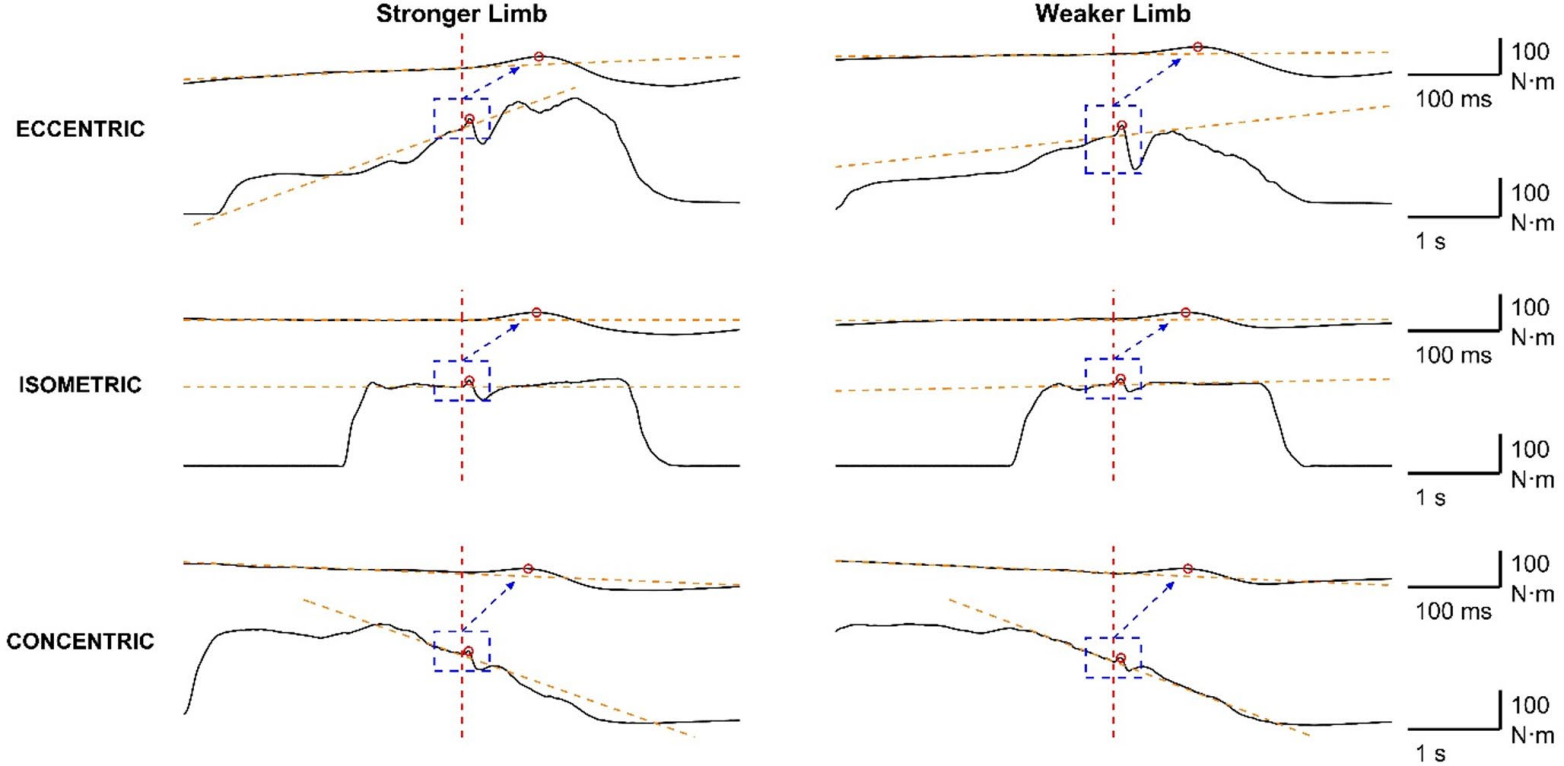
Superimposed twitch technique applied during voluntary knee extensor contractions in the stronger (left column) and weaker (right column) limbs of one representative healthy participant, across three contraction types: eccentric (top row), isometric (middle row), and concentric (bottom row). For each condition, the upper panel shows an expanded view of the superimposed twitch torque around the neurostimulation onset, and the lower panel shows the full torque-angle curve. The dashed red vertical line indicates the timing of the superimposed doublet electrical stimulus. The orange dashed line represents the linear regression fitted to the torque signal over the 250 ms preceding the neurostimulation. The red circle indicates the superimposed twitch torque. The blue dashed rectangle in the lower panel delineates the time window displayed in the upper panel

Torque was filtered using a low-pass 4th order Butterworth filter at a cutoff frequency of 10 Hz. For each contraction type the torque was measured on the two limbs to a knee angle of 30 degrees (T_30_). To measure the superimposed doublet torque response, we used the method developed by Babault and colleagues in 2001 (Babault et al. 2001). With this method we estimated the torque values, without the disruption caused by neurostimulation, with a linear regression calculated over a window starting 250 ms before the neurostimulation and ending at it. The superimposed doublet torque was then calculated as the Euclidean distance between the maximum torque value and the corresponding point on the regression line, which was extended over a 200 ms window following stimulation. This method is illustrated for one representative subject in Fig. 1. The same analysis and calculation method were used to determine the potentiated resting doublet-evoked torque. To avoid overestimating the superimposed doublet torque during stimulation when the subject was not generating their maximal torque for that contraction, we normalized the superimposed doublet torque by dividing the pre-stimulation torque by the maximum torque observed across the eight trials, keeping the angle (30°), type of contraction, and limb consistent. For this, we used the following equation (Beltman et al. 2004):

$$\frac{{1 - {\mathrm{D}}*\left( {{\mathrm{Tb}}/{\mathrm{Tmax}}} \right)*100}}{{{\mathrm{TTW}}}}$$

where D is the amplitude of the superimposed doublet torque measured during maximal voluntary contractions, Tb is the voluntary torque collected just before the superimposed doublet, Tmax is the maximum torque observed across the eight trials keeping the angle (30°), type of contraction, and limb consistent, and TTW is the amplitude of the potentiated doublet torque measured at rest.

#### EMG activity and M-wave

For three heads of the quadriceps (VM, VL and RF) the EMG signals were band pass filtered using a 4th order Butterworth filter (20–500 Hz). The filtered EMG signal was then quantified as Root Mean Square (RMS) over a 500 ms window during each maximal contraction, starting 510 ms before the delivery of the stimulation. For each muscle, the compound muscle action potential (M-wave) was recorded following stimulation and quantified as the peak-to-peak amplitude, with the mean value computed from three trials per muscle. This M-wave amplitude was subsequently used to normalize the RMS EMG signal, expressed as the ratio of RMS to M-wave (RMS/M-wave), with contraction types and limbs matched for each measurement.

#### Calculation of asymmetries

The stronger or weaker limb were identified based on the torque values. For each limb, the sum of torque produced at 30° was calculated across the three contraction types, and the limb with the higher summed value was defined as the stronger limb. This limb classification was then used to compute asymmetries in torque, voluntary activation, resting doublet-evoked torque, and EMG activity. We then calculated the asymmetries with the Asymmetry Index: [(stronger limb – weaker limb) / stronger limb] * 100 for these four variables. To compare different asymmetry metrics, additional indices were calculated: the Bilateral Asymmetry Index: [(stronger limb – weaker limb) / (stronger limb + weaker limb] * 100; the Symmetry Angle: [45 – arctan (weaker limb) / (stronger limb) / 90 * 100; and the Limb Symmetry Index (LSI): (weaker limb) / (stronger limb) * 100, all computed for torque.

A secondary analysis examined asymmetries between the dominant and non-dominant limbs. Limb dominance was defined by the participants’ answer to the question: ‘If you had to shoot a target, which leg would you use to shoot the ball’. In this analysis, asymmetry was again expressed using the Asymmetry Index: AI (%) = (D – ND) / (D) where D and ND denote values from the dominant and non-dominant limbs, respectively.

### Statistical analysis

All data are presented as mean ± SD. Normality and homogeneity of variance were assessed using the Shapiro–Wilk and Levene tests, respectively. When these assumptions were not met, appropriate non-parametric procedures were applied. Bayesian analyses were additionally performed to quantify the evidence for the null hypothesis, particularly in cases where frequentist tests did not reach significance (*p* > 0.05), with Cauchy priors on standardized effect sizes (*r* = 0.5 for fixed effects, *r* = 1.0 for random effects, and *r* = 0.354 for covariates; default Bayes Factor settings).

A two-way repeated measures ANOVA with contraction type (isometric vs. concentric vs. eccentric) and limb (stronger vs. weaker) as within-subject factors was used to analyse torque, voluntary activation level (VAL), potentiated doublet at rest, and EMG activity (RMS EMG/M). This approach allowed comparison of stronger and weaker limbs and evaluation of main and interaction effects of contraction type. Statistical significance was set at *p* < 0.05 and significant effects were followed up with Tukey’s HSD post hoc tests.

Complementary analyses were conducted to assess potential fatigue effects during the experimental session. Fatigue monitoring was achieved by comparing within-series torque variability, expressed as the CV across the eight MVCs, between the first and last series performed for each limb and participant. These CV values were analyzed using two‑tailed paired‑samples t‑tests to determine whether torque variability significantly differed between the first and last series, as torque CV is known to increase with fatigue (Missenard et al. 2009).

To compare the different asymmetry indices on a common scale, each index was standardized within participants using Z-score transformation (Z = [individual value − mean] / SD). A two-way repeated-measures ANOVA with contraction type (isometric, concentric, eccentric) and asymmetry index (asymmetry index, bilateral asymmetry index, lateral symmetry index, symmetry angle) as within-subject factors was then performed.

Torque asymmetry was examined at the individual level using intra-individual variability. For each contraction type, inter‑limb asymmetry, computed either from stronger–weaker or dominant–non‑dominant comparisons, was considered present when it exceeded the mean CV derived from eight trials on the stronger (or dominant) and eight trials on the weaker (or non‑dominant) limb, as described by Exell et al. (2012). This yielded a binary indicator of significant asymmetry (present/absent) for each participant and contraction type. Given that these data involved repeated within-subject binary observations across contraction types, the proportion of significant asymmetries was compared using Cochran’s Q test for related samples.

Torque asymmetries and EMG activity during anisometric contractions were further evaluated using angle-specific analysis. Statistical Parametric Mapping (SPM) was used to compare torque and EMG activity vectors at each measured point across the range of motion (Pataky 2010). The convergence of parametric and non-parametric SPM results supported the normality assumption. The scalar output statistic SPM{F} was computed over for the ROM (0° to 80°) and paired-sample t-tests SPM{t} were subsequently performed. For these analyses, each data set was normalized by its maximum value to emphasize relative torque profiles rather than absolute magnitudes. For the EMG activity, a sliding window of 50 ms was employed, with the RMS calculated for each window.

The association between torque asymmetry and both activation-level and doublet-evoked torque asymmetries was first analyzed using a linear mixed-effects model with torque asymmetry as the dependent variable and asymmetries computed between stronger and weaker limbs. Activation asymmetry and doublet asymmetry were entered as continuous covariates, and contraction type was included as a fixed factor. To account for between-subject variability, participant-specific random intercepts were specified, and model performance was summarized using marginal and conditional R². As a complementary sensitivity analysis, the same model was repeated with asymmetries computed between dominant and non-dominant limbs, to avoid relying solely on a torque-based limb classification that could condition the analysis on the outcome.

## Results

### Torque

**Fig. 2:**
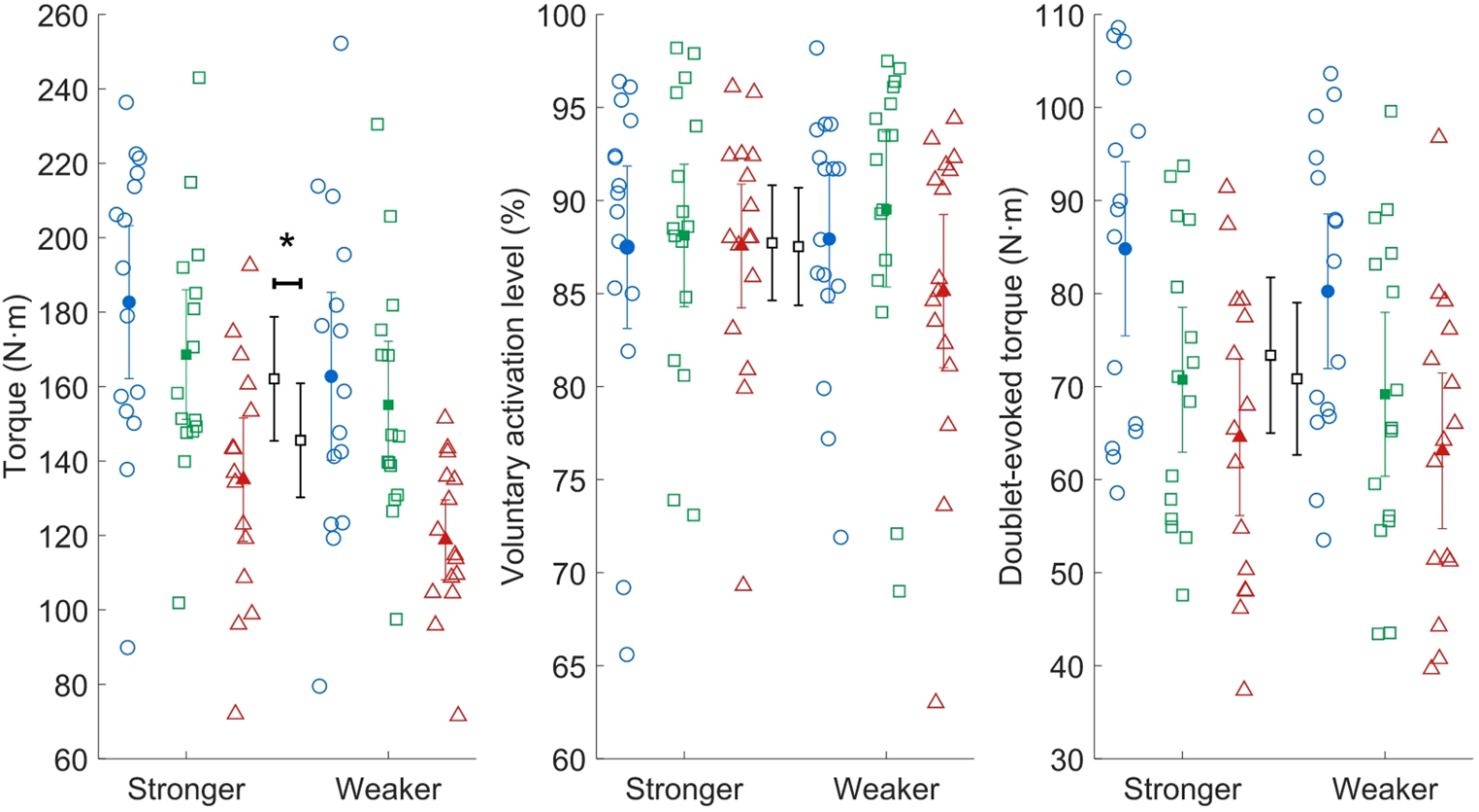
Each panel’s figure shows maximal voluntary torque, voluntary activation level (VAL), doublet evoked torque respectively. The white shape represents individual data, with blue circles represent eccentric contractions, green squares represent isometric contractions, and red triangles represent concentric contractions. The dark-colored symbols represent the mean of each type of contraction. White squares in the middle of each panel indicate the mean values for the strong and weak limbs. Asterisks indicate significant differences with *p* < 0.05, and the lower and upper error bars indicate the 95% confidence interval, respectively

The two-factor repeated-measures ANOVA on torque values revealed significant main effects of limb (F(1,14) = 29.40, *p* < 0.001, η²*p* = 0.67; Fig. 2) and contraction type (F(2,28) = 30.96, *p* < 0.001, η²*p* = 0.68). No limb × contraction type interaction was detected (*p* > 0.05). Post hoc comparisons indicated that, regardless of contraction type, torque was greater in the stronger than in the weaker limb (t(14) = 5.42, *p* < 0.001; Fig. 2), acknowledging that designating the “stronger” limb post hoc may introduce bias. Across limbs, isometric torque exceeded concentric torque (t(14) = 5.78, *p* < 0.001), eccentric torque exceeded concentric torque (t(28) = 7.39, *p* < 0.001), and isometric and eccentric torque did not differ significantly (*p* > 0.05). To verify that the potential influence of fatigue on force production capacity did not confound the results, complementary analyses were conducted. Torque CV remained stable across repeated series, with no significant difference between the first and last sets of eight contractions on the same limb (t(29) = 0.761, *p* = 0.453, BF₁₀ = 0.254).

Mean asymmetry index values were 7.8% (± 8.4) for isometric, 11.3% (± 10.2) for eccentric, and 9.7% (± 14.1) for concentric conditions. At the individual level, 27% (4/15) of participants displayed asymmetry during isometric contractions, 60% (9/15) during concentric contractions, and 53% (8/15) during eccentric contractions. No difference in asymmetry prevalence was observed across contraction types, as indicated by Cochran’s Q test (Q(2) = 4.0, *p* = 0.135). When applying an arbitrary threshold of 15% (Lockie et al. 2016) commonly referenced in the literature, asymmetry prevalence decreased to 13.3% (2/15) for isometric contractions and 33.3% (5/15) for both eccentric and concentric contractions.

Finally, a two-factor repeated-measures ANOVA conducted on Z scores revealed no significant main effects of index or contraction type (both *p* > 0.05), indicating that asymmetry magnitude was comparable across indices.

Furthermore, when strength was compared between the dominant and non-dominant limbs in a second analysis, the two-way repeated-measures ANOVA revealed no significant difference between limbs (*p* > 0.05). When torque asymmetries (dominant vs. non-dominant) were contrasted with individualized torque CV, 6 participants in the isometric condition, 9 in the eccentric condition, and 8 in the concentric condition showed torque asymmetries exceeding their individual variability threshold.

**Fig. 3:**
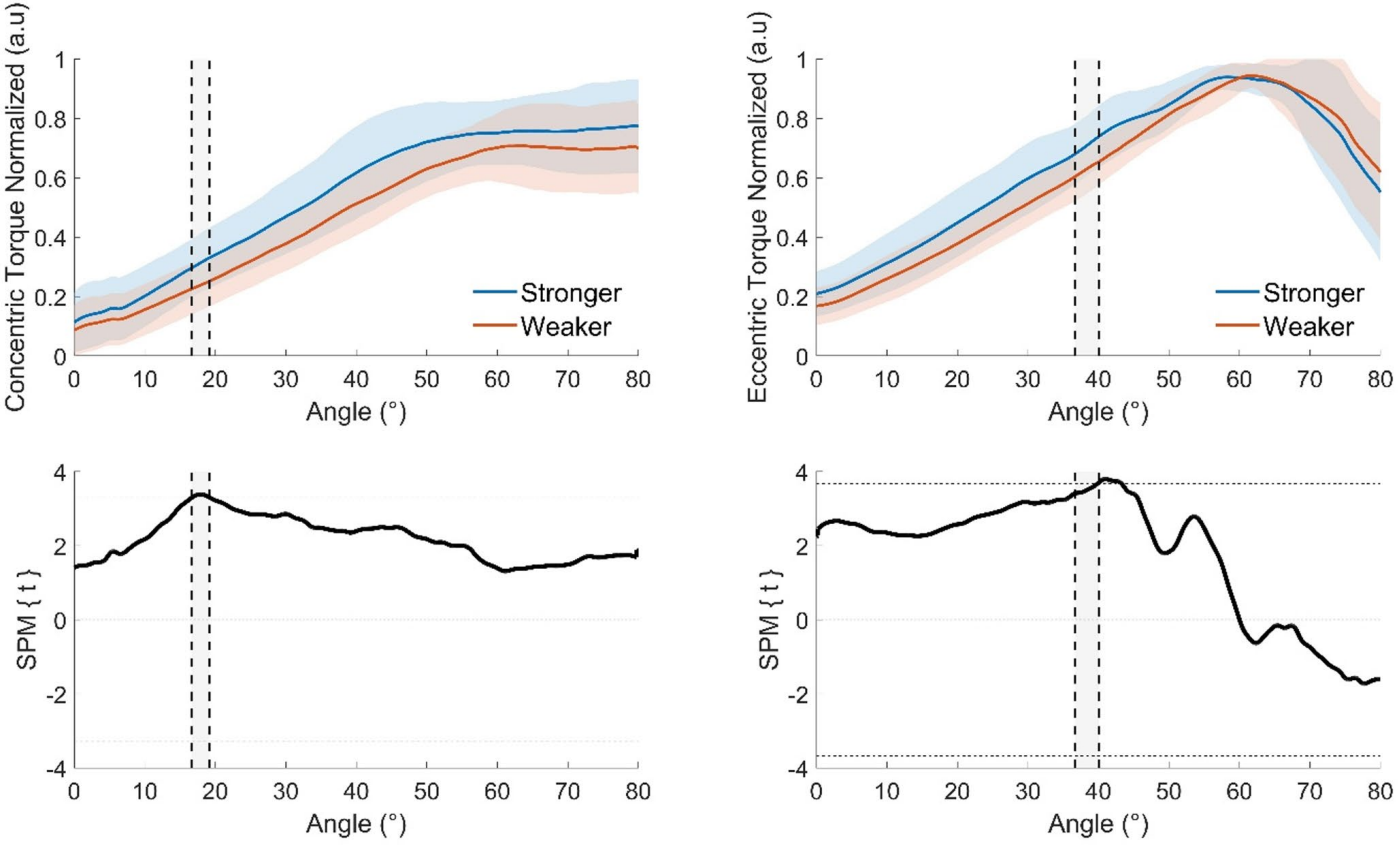
Normalized knee extensors angle-specific torque analysis, between the stronger limb (dark gray) and the weaker limb (light gray), in concentric contractions (left graph) and eccentric contractions (right graph). The top panels display the mean (± SD) normalized torque across the 15 subjects as a function of knee angle, and the bottom panels show the corresponding statistical parametric maps (SPM) for the paired t-tests between limbs. The shaded gray bands indicate the angle ranges where the differences between stronger and weaker limbs are statistically significant (*p* < 0.05)

Angle-specific torque analysis showed that, during concentric contractions, torque of the stronger limb was significantly higher than that of the weaker limb between 13° and 16° of knee flexion (*P* < 0.05; Fig. 3). During eccentric contractions, this difference was significant between39° and 43° of knee flexion (*P* < 0.05; Fig. 3).

### EMG activity, voluntary activation, and doublet-evoked torque

The two-factor repeated-measures ANOVA revealed no significant difference in voluntary activation between the stronger and weaker limbs (*p* > 0.05), with Bayesian analyses providing evidence in favor of the null hypothesis (BF₁₀ = 0.21). Similarly, voluntary activation did not differ across contraction types (*p* > 0.05; BF₁₀ = 0.30). Doublet-evoked torque was also comparable between limbs (*p* > 0.05; BF₁₀ = 0.28); however, a significant main effect of contraction type was observed (F(2,28) = 93.50, *p* < 0.001, η²*p* = 0.87). Post hoc analyses indicated greater doublet-evoked torque during eccentric contractions compared with isometric (t(14) = 11.43, *p* < 0.001) and concentric contractions (t(14) = 11.59, *p* < 0.001), and higher torque during isometric than concentric contractions (t(14) = 4.31, *p* < 0.05).

No significant differences were found in EMG/M RMS of the VM, VL and RF between limbs or across contraction types (*p* > 0.05). Bayesian analyses supported the absence of differences for VM and RF (BF₁₀ = 0.25 and 0.29, respectively), but not for VL (BF₁₀ = 8.30). In addition, SPM analyses revealed no significant differences in EMG activity for VM, VL or RF across the three contraction types.

**Fig. 4:**
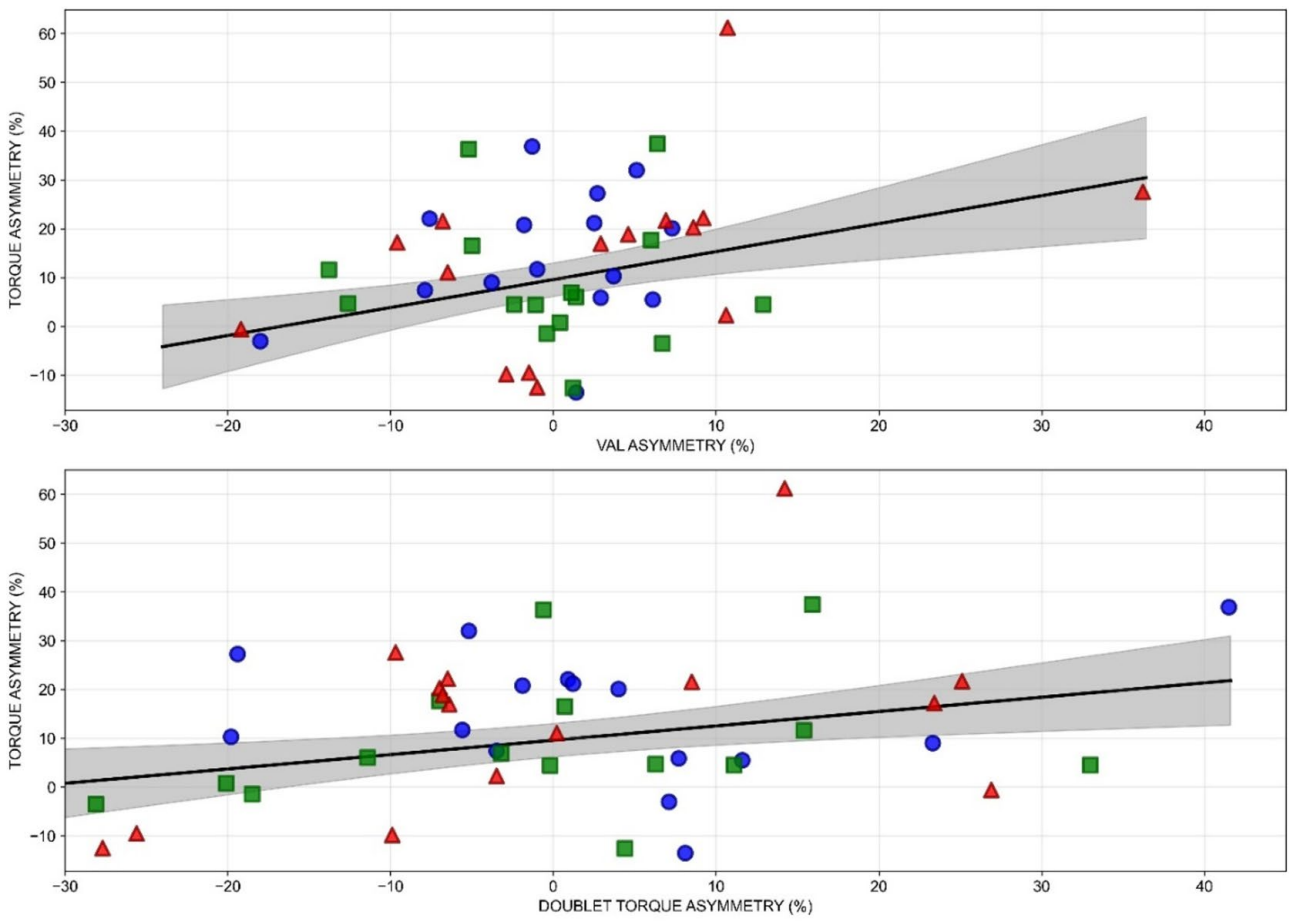
Mixed-effects linear model describing the covariation between torque asymmetry and neuromuscular asymmetries. Asymmetries were defined using stronger versus weaker limbs. Top panel: covariation between torque asymmetry and voluntary activation level (VAL) asymmetry. Bottom panel: covariation between torque asymmetry and doublet evoked torque asymmetry. Blue circles, green squares, and red triangles represent eccentric, isometric, and concentric contractions, respectively. Solid lines indicate the fitted linear mixed-effects model, and shaded areas represent the 95% confidence interval of the fit

Linear mixed model analysis indicated that asymmetries in activation level (estimate = 0.57, *p* = 0.001) and doublet‑evoked torque (estimate = 0.29, *p* = 0.007) were significant predictors of torque asymmetry (Fig. 4), together explaining 29% of its variance via the fixed effects (marginal R² = 0.29; conditional R² = 0.37) when asymmetries were defined using stronger versus weaker limbs. In addition, these covariations were not significantly influenced by contraction type (*p* = 0.746). As a complementary sensitivity analysis, we repeated the model with asymmetries defined with respect to dominant versus non-dominant limbs; this produced consistent findings, with activation-level (estimate = 0.77, *p* = 0.001) and doublet-evoked torque asymmetries (estimate = 0.38, *p* = 0.040) again significantly predicting torque asymmetry and explaining 20% of its variance via the fixed effects (marginal R² = 0.20; conditional R² = 0.63), and these covariations were also not significantly influenced by contraction type (*p* = 0.551).

## Discussion

In the present study, we investigated the contribution of neuromuscular mechanisms to inter-limb strength asymmetries during isometric, concentric, and eccentric contractions. Overall, the findings indicate a significant difference in strength between the stronger and weaker limbs, whereas no corresponding differences were observed in neuromuscular parameters, including activation level, EMG activity, and resting doublet-evoked torque. In contrast to the limb-to-limb comparisons, torque asymmetry was positively associated with asymmetries in doublet-evoked torque and activation level, independently of contraction type.

### Methodological considerations for the measurement of strength asymmetries

This study reveals significant differences in strength values between stronger and weaker limbs, in healthy young adults, with mean asymmetry magnitudes for isometric contractions around 7–8%, and for eccentric and concentric contractions ranging between 10 and 12%. These results align with previous studies in similar populations reporting asymmetries of 10–12% for concentric and 7–12% for eccentric contractions (Jones and Bampouras 2010; Thomas et al. 2017). Complementary analyses confirmed that these strength asymmetries were not affected by fatigue, as torque CV across MVCs did not differ between the first and last series, indicating no detectable fatigue effect at the session level. Nevertheless, while torque asymmetries in strength are not always observed in healthy individuals, the inconsistent reporting across previous studies may be attributed to various methodological differences, including (i) the choice of the reference limb, (ii) whether an angle-specific analysis or a single angle was used, (iii) the selection of the asymmetry index, and (iv) the threshold for determining significant asymmetry (Parkinson et al. 2021). First, our results indicate that no differences are observed between the limbs when comparing the dominant and non-dominant limbs, which has also been demonstrated in other studies using this methodology (Daneshjoo et al. 2013; González-Ravé et al. 2014). In contrast, the comparison between stronger and weaker limbs shows significant differences in our study, highlighting the importance of the choice of reference limb when measuring asymmetries, as it can influence the detection of strength asymmetries. Nevertheless, the comparison between stronger and weaker limbs can be challenging, as the direction of asymmetries (positive or negative) may vary depending on the measurement conditions (for example, between contraction types), especially when subtle asymmetries are present. Once the reference limb is determined, asymmetries can be calculated at a specific angle or through range of motion specific analysis (Eustace et al. 2022). In our study, asymmetries are more prominent at shorter muscle lengths in concentric contractions and at medium lengths in eccentric contractions. These results are obtained through a Statistical Parametric Mapping (SPM) analysis, which also has limitations regarding statistical sensitivity. This suggests that, unlike single-angle analyses, an SPM analysis provides a more comprehensive evaluation by targeting specific angular sectors of asymmetry depending on the type of contraction (Kocak et al. 2023). Notably, results at 30° may not align with the range identified by SPM, likely reflecting differences in statistical sensitivity between the methods. Accurately quantifying these asymmetries can be achieved through various indices of asymmetry described in the literature (Parkinson et al. 2021). Interestingly, in our study, the choice of index did not significantly influence the detection of asymmetries, as similar results were obtained regardless of the index used. This indicates that, while we compared various calculation methods - including the index of symmetry (LSI), the index of asymmetry (asymmetry index and bilateral asymmetry index), and the symmetry angle, which offers a geometric representation of the direction and magnitude of asymmetry - the choice of method does not appear to significantly affect the detection of asymmetries. In contrast, the selection of the appropriate reference limb remains critically important. Finally, to assess whether an individual asymmetry is significant, we compared the subject’s asymmetry value to the CV between trials under the same condition (angle and contraction type). Our results in healthy individuals indicate that using individualized thresholds increases the detected prevalence of asymmetry relative to the commonly used 15% cut-off (Lockie et al. 2016) across all contraction types; however, these findings depend on intra-individual variability and may differ in pathological populations. In this context, the threshold should be regarded as a pragmatic heuristic rather than a strict physiological cut-off that clearly separates “relevant” from “non-relevant” strength asymmetry in healthy individuals. Our findings underscore the importance of methodological choices, particularly the selection of the reference limb and calculation threshold, in accurately detecting strength asymmetries. Overall, these factors highlight and confirm the need for standardized approaches in future research to ensure consistent and reliable measurements of interlimb strength asymmetries.

### Neuromuscular parameters involved in strength asymmetries

Although the methods for measuring strength asymmetries in healthy individuals have yet to be standardized, understanding the neuromuscular mechanisms involved in a strength asymmetry can help therapists target strategies to restore these asymmetries. One of the mechanisms involved in strength asymmetries may be an asymmetry in the peripheral muscle capacities of one limb compared to the other (Lepley et al. 2020). These asymmetries can be attributed to sports demands, with greater muscle volumes in the dominant upper limb of tennis players for example (Sanchís-Moysi et al. 2010), or associated with the hypoactivity resulting from an ACL rupture, leading to deficits in cross-sectional area (Norte et al. 2018). In comparison, our results for the lower limbs in healthy subjects showed no difference in doublet‑evoked torque between the stronger and weaker limbs, indicating no excitation-contraction coupling deficit, although this measure does not account for other peripheral factors such as muscle size, tendon and aponeurosis mechanical properties, or moment arm differences, which were not assessed in the present study. This lack of asymmetry likely reflects the lower limbs’ largely symmetrical loading in daily activities, which limits peripheral asymmetries, unlike the upper limbs exposed to more unilateral demands. The neuromuscular parameters contributing to torque asymmetries extend beyond peripheral muscular factors; neural activation asymmetry may also explain strength asymmetries (Urbach et al. 2001). To investigate this, we compared the voluntary activation levels and the EMG activity between the stronger and weaker limbs. We observed no activation level asymmetries at 30° across all contraction types. This aligns with prior studies reporting no activation asymmetries in healthy individuals during isometric contractions at 60° and 30° (de Ruiter et al. 2010; Pietrosimone et al. 2012). We also observed no EMG differences between the stronger and weaker limbs across all contraction types at 30°. This finding is consistent with previous reports showing no differences in EMG activity between dominant and non-dominant limbs in the quadriceps (Yılmaz and Kabadayı 2022) and the tibialis anterior during isometric contractions (Petrovic et al. 2021). Furthermore, when we extended the EMG analysis with SPM across the full joint-angle range, we likewise observed no asymmetries. In contrast, our analysis of activation levels was restricted to a single joint angle (30°). Since activation level may vary with joint angle (Doguet et al. 2017), asymmetries in voluntary activation that occur at different muscle lengths may therefore have gone undetected. However, Pietrosimone et al. (2012) assessed voluntary activation at 30°, 70°, and 90° during isometric contractions and likewise found no between-limb asymmetry. Taken together, these results suggest that measurement angle - and thus muscle length - may not materially influence neural activation asymmetries, but further studies are needed to confirm this across wider angle ranges during anisometric contractions. While most studies examine neuromuscular mechanisms during voluntary isometric contractions (de Ruiter et al. 2010; Petrovic et al. 2021; Pietrosimone et al. 2012; Yılmaz and Kabadayı 2022), we aimed to extend the analysis to anisometric conditions, as they are more representative of clinical assessments (Xergia et al. 2013). Similar to the isometric condition, our results do not show any significant differences between the stronger and weaker limbs in concentric and eccentric contractions, neither in terms of voluntary activation level nor in EMG activity. Nevertheless, the absence of between-limb differences in neuromuscular parameters should be interpreted in the context of both the modest torque asymmetries observed in healthy subjects, and the reliability and variability characteristics of the neuromuscular parameters. Prior studies indicate that voluntary activation and doublet-evoked torque generally exhibit low variability and high repeatability (Todd et al. 2004). By contrast, in our data, RMS EMG showed higher within‑trial variability (CV ranging from 12 to 19% across limbs, muscles and contractions types) which may have reduced our sensitivity to detect small between‑limb EMG differences.

Under isometric conditions, analyses of covariation rather than simple between-limb comparisons have shown that asymmetries in activation level and in rested doublet-evoked torque positively covary with torque asymmetries in both healthy individuals and patients with ACL injury (Maffiuletti et al. 2016). Our results confirm a significant positive covariation between activation level asymmetries and rested doubled evoked potential asymmetries with torque asymmetries, with the particularity of incorporating anisometric contractions into the mixed linear model. Importantly, these positive covariations were not influenced by the type of contraction suggesting that the results obtained in isometric contractions could be applicable to anisometric conditions, although this non-significant interaction should be interpreted with caution given the modest sample size (*n* = 15) and the absence of readily available power estimation for this mixed-model design. To further assess the robustness of these findings, we repeated the mixed-model analysis using an independent definition of limb (dominant vs. non-dominant), thereby reducing the risk of conditioning the analysis on torque-based limb classification. This complementary analysis yielded comparable associations between activation-level and doublet-evoked torque asymmetries and torque asymmetry, supporting the robustness of these covariation findings and indicating that they remain independent of contraction type. Consistently, the marginal R² values were similar across both models (0.20 vs. 0.29), by contrast, the conditional R² was higher in the dominant–non-dominant model (0.63 vs. 0.37), reflecting differences in how between-subject variability was represented by the random intercepts rather than substantive changes in the fixed effects. Regardless the mixed linear model, the estimated value for neural activation was found to be higher than that of muscular parameters (rested doublet-evoked torque). This suggests that, in our subjects, changes in neural activation may have a more significant influence on torque variations. These findings have practical implications, as understanding the role of neuromuscular parameters in torque asymmetries can enable therapists to adapt their muscle strengthening protocols more efficiently. If asymmetries are primarily associated with deficits in peripheral muscle parameters, an emphasis on hypertrophy-oriented programs would be appropriate (Welling et al. 2019). Conversely, if they are linked to impaired neural activation, targeted resistance training modalities—such as high intensity loading, prolonged time under tension, or maximal intended velocity **-** and also protocols using EMG biofeedback could be effective in enhancing muscle recruitment (Balshaw et al. 2016; Jenkins et al. 2017; Needle et al. 2017). Furthermore, the model explains only 29% of the variation in torque asymmetries in young healthy men, suggesting that contribution of additional neuromuscular mechanisms such as between-limb differences in muscle coactivation (Avrillon et al. 2018) or muscle cross-sectional area (Norte et al. 2018), remain to be identified.

## Conclusion

In summary, this study in healthy men adults demonstrated moderate strength asymmetries between stronger and weaker limbs at 30° knee flexion across isometric, concentric, and eccentric contractions. Despite these torque differences, we could not detect concurrent significant differences between limbs for neuromuscular parameters regardless of the contraction type, likely due to the small magnitude of asymmetries in this healthy cohort, and the inherent variability of these neuromuscular measures. Nevertheless, neuromuscular asymmetries positively covaried with torque asymmetries and this association was not modulated by contraction type. These findings highlight the need to determine whether similar relationships between neuromuscular parameters and torque asymmetries exist in clinical populations, and whether targeting these neuromuscular deficits can effectively reduce strength imbalances.

## Abbrevations

ACL: Anterior cruciate ligament
ANOVA: Analysis of variance
CV: Coefficient of variation
EMG: Electromyographic
MDC: Minimal detectable change
RMS: Root mean square
VM: Vastus Medialis
VL: Vastus Lateralis
RF: Rectus Femoris
